# Asymmetric LSCF Membranes Utilizing Commercial Powders

**DOI:** 10.3390/ma13030614

**Published:** 2020-01-30

**Authors:** Paolo Fedeli, Francesca Drago, Falk Schulze-Küppers, Stefan Baumann

**Affiliations:** 1 Ricerca sul Sistema Energetico—RSE SpA, Strada Torre della Razza, I-29122 Piacenza, Italy; 2 Ricerca sul Sistema Energetico—RSE SpA, Via Rubattino 54, I-20134 Milan, Italy; francesca.drago@rse-web.it; 3 Forschungszentrum Jülich GmbH, Institute of Energy and Climate Research, Materials Synthesis and Processing (IEK-1), 52425 Jülich, Germany; f.schulze@fz-juelich.de (F.S.-K.); s.baumann@fz-juelich.de (S.B.)

**Keywords:** oxygen transport membrane, tape casting, ceramic powder characterization, asymmetric membrane manufacturing

## Abstract

Powders of constant morphology and quality are indispensable for reproducible ceramic manufacturing. In this study, commercially available powders were characterized regarding their microstructural properties and screened for a reproducible membrane manufacturing process, which was done by sequential tape casting. Basing on this, the slurry composition and ratio of ingredients were systematically varied in order to obtain flat, crack-free green tapes suitable for upscaling of the manufacturing process. Debinding and sintering parameters were adjusted to obtain defect-free membranes with diminished bending. The crucial parameters are the heating ramp, sintering temperature, and dwell time. The microstructure of the asymmetric membranes was investigated, leading to a support porosity of approximately 35% and a membrane layer thickness of around 20 µm. Microstructure and oxygen flux are comparable to asymmetric La_0.6_Sr_0.4_Co_0.2_Fe_0.8_O_3−δ_ (LSCF) membranes manufactured from custom-made powder, showing an oxygen flux of > 1 mL⋅cm^−2^⋅min at 900 °C in air/Ar gradient.

## 1. Introduction

With a production of more than 100 million tons per year, oxygen is one of the largest chemical commodities worldwide [1]. Pure O_2_ is used in a large variety of industrial applications, such as the production of glass, cement, ceramics, chemicals, and petrochemicals. Therefore, the generation of high purity O_2_ by low-cost and environmental-friendly processes is an important issue.

So far, cryogenic distillation and pressure swing adsorption have been mainly applied for the production of commercial oxygen. However, such technologies require both high energy consumption and operating costs. An attracting alternative is oxygen separation from air by means of ceramic oxygen transport membranes (OTMs), thanks to the low efficiency losses compared with traditional processes [2]. OTMs are gastight structures made of mixed ionic electronic conducting (MIEC) materials, where oxygen ions transport can take place through oxygen vacancies in the crystal lattice. Mixed ionic–electronic conduction was initially reported in solid oxide materials by Takahashi et al. [3] and MIEC solid oxides were first applied for oxygen separation in the early 1980s [4,5]. After the seminal work of Teraoka et al. [6], perovskite oxides La_1−x_Sr_x_Co_1−y_Fe_y_O_3−δ_ emerged as a promising class of MIEC materials for OTMs. Since then, an extensive investigation activity has been conducted on the composition La_0.6_Sr_0.4_Co_0.2_Fe_0.8_O_3−δ_ (LSCF), which has been identified as model material, since it is one of the best investigated mixed ionic electronic conducting materials with a sufficiently high oxygen flux and mechanochemical stability for oxygen [7,8,9,10,11,12,13,14].

According to the Wagner theory of transport in solid oxide [15], the oxygen bulk transport through a dense MIEC layer is hampered by the layer thickness. Therefore, high permeation rates can be achieved by reducing the membrane thickness. The desired mechanical stability is usually obtained by an asymmetric structure, where a thin dense layer is supported on a thick porous layer [7,12,16].

Tape casting is a promising technology for the manufacturing of solid oxide OTMs on the industrial scale, as a well-established technique for the high-volume and cost-effective production of planar ceramic structures [17,18,19]. Tape casting entails several advantages, such as the accurate control of the layer thickness in a wide range and the possibility to cast large area structures, which are essential for the scale-up of the membrane technology. Moreover, multilayered structures can be fabricated by using sequential tape casting, i.e., by directly casting different layers one on top of the other. This approach limits as much as possible the number of manufacturing steps, with significant advantages in terms of the duration and costs of the production process. For these reasons, the preparation of asymmetric OTMs by tape casting has been widely investigated in the last years [7,12,20,21,22].

Nevertheless, tape casting remains a complex technique, and the successful manufacturing of components especially depends on the formulation of the correct slurry composition, which is, in turn, dramatically affected by the ceramic powder properties [17,23,24]. Therefore, the slurry composition must be adjusted according to the used powders in order to obtain a proper slurry viscosity and avoid the formation of defects such as pinholes, cracks, or bending during the drying of the cast tapes [25]. Most of the commercially available ceramic powders are suitable for different ceramic manufacturing technologies, e.g., extrusion, but not for tape casting. Specifically designed powders for tape casting are usually custom-produced at the lab scale and are not available on large industrial quantities. 

In the present work, five commercially available LSCF powders were characterized and compared with a custom powder, manufactured as Research&Development (R&D) Batch by Solvay, Belgium, specifically for tape casting and successfully used to manufacture asymmetric membranes by tape casting in the FP7 EU project GREEN-CC [26]. The most promising commercial powder was identified and used for slurry development. The slurry composition was identified starting from an already published reference recipe for the manufacturing of asymmetric membranes [12,26]. The debinding process for tape cast membranes was optimized accordingly, and the microstructure of the sintered membranes was analyzed and compared to the LSCF membrane reported in literature. The main goal was to investigate a reproducible production of membranes by tape casting in dependence of the LSCF powder properties and morphology.

## 2. Materials and Methods 

Five commercial LSCF powders were purchased by different manufacturers and labeled as A, B, C, D, and E, respectively. Powders A and B were purchased from FuelCellMaterials (Lewis Center, OH, USA), powder C from CerPoTech AS (Trondheim, Norway), and powders D and E were purchased from Praxair Inc. (Danbury, CT, USA). The choice was made to have powders with different specific surface area and mean grain size values, as reported on the certificates of analysis provided by the manufacturers. The powders were selected among those available on market with an appropriate grain size distribution for tape casting, in the range of 1–10 μm. Powders with larger particles, i.e., specifically designed for spray-coating processes, were not considered. 

The five powders were characterized in terms of their morphology, crystallographic phase, chemical composition, sintering behavior, and permeation. The properties of the powders were compared to those of a custom powder, which was manufactured as an R&D batch specifically for tape casting (Solvay, Brussels, Belgium). Two out of five powders, C and D, were used to prepare organic-based ceramic slurries for the manufacturing of asymmetric green layers by sequential tape casting followed by a debinding and sintering process. The properties of the manufactured membranes, in terms of microstructure and oxygen permeation, were compared with those of membranes manufactured by using the Solvay custom powder.

### 2.1. Powders Characterization

Powders morphology was examined in a Scanning Electron Microscope (SEM FEG Tescan MIRA3, Brno, Czech Republic). The particle size distribution (PSD) and specific surface area of the powders were investigated by laser diffraction using a Malvern Mastersizer 3000 granulometer (Malvern, UK) equipped with a Hydro EV humid dispersion unit and by nitrogen adsorption method (Brunauer–Emmett–Teller analysis) using an Areameter from Ströhlein GmbH (Düsseldorf, Germany) with Nitrogen as measurement gas at a temperature of −196 °C, respectively.

To investigate the phase composition, X-ray diffraction (XRD) measurements were performed in Bragg–Brentano configuration using a Bruker D4 Endeavor diffractometer (Karlsruhe, Germany) operating with a Cu tube (40 kV and 40 mA). The stoichiometry was examined by inductively coupled plasma optical emission spectroscopy (ICP-OES), using a Agilent ICP 700 spectrometer (Santa Clara, CA, USA) with a precision of 1–3% for major elements. 

The sintering behavior was investigated by differential dilatometry using an NETZSCH DIL 402 C dilatometer (Selb, Germany). Measurements were performed on uniaxial dry-pressed powder cylindrical-shaped samples with a diameter of 8 mm, obtained by applying a pressure of 140 MPa. The samples were treated in air up to a temperature of 1300 °C with heating and cooling rates of 5 K/min and a dwell time of 5 h. 

The oxygen permeation properties of the five different powders were compared by performing oxygen permeation measurements on sintered powder discs with thickness of 1 mm and diameter of 14.7 mm, obtained by uniaxial dry-pressing at 40 MPa and sintering at 1300 °C for 5 h. Measurements were carried out in air/Ar gradients in the permeation quartz cell described in [20], in the temperature range of 750–1000 °C and at atmospheric pressure. A constant flow rate of 250 mL/min of ambient air and 50 mL/min of Ar were fed as the feed and sweep gas, respectively. The sealing between the samples and the permeation cell was ensured at about 1030 °C by placing the samples between two gold rings, and it was considered acceptable when the ratio between the oxygen flow through the defects and the total oxygen flux was lower than 1%. The oxygen permeation was determined by measuring the oxygen concentration in the sweep gas stream by a mass spectrometer (Omnistar, Pfeiffer Vacuum, Asslar, Germany). The membrane gas tightness was verified during the permeation tests by continuously monitoring the N_2_ concentration in the permeate. The maximum leakage value of oxygen was about 5.5% at the lowest temperature.

The experimental error estimated on the O_2_ flux is approximately 5%. The experimental setup described above was also used to characterize the manufactured asymmetric LSCF membranes.

The results of these characterizations were compared to the reference LSCF powder given in [26].

### 2.2. Membrane Preparation

Slurries for the membrane layer and support layer (referred to as “membrane slurry” and “support slurry”, respectively) were prepared separately, starting in both cases from existing recipes optimized for other ceramic powders [12,19]. A two-step procedure was followed. First, ceramic powders were dispersed in an ethanol and methyl ethyl ketone (MEK) mixture using Nuosperse FX9086 (Elementis Specialties, Inc., London, UK) as the dispersing agent. After that, polyvinyl butyral (Butvar® PVB B-98, Solutia Inc., St. Louis, MO, USA) as binder, Solusolv 2075 (Solutia Inc.) as plasticizer I, and PEG400 (Merck KGaA, Darmstadt, Germany) as plasticizer II were added to the slurry to provide mechanical stability and flexibility to the green tape. Only in the case of support slurry, corn starch (Cargill C-Gel, Krefeld, Germany) was added in the first step of the preparation as the pore-forming agent, in order to obtain a percolating porous structure after burn out. Details on the slurry preparation procedures are described in [19,27]. 

The content of additives (i.e., binder, plasticizer I and II) of membrane and support slurries was adjusted according to the ceramic powders’ properties. 

**Figure 1:**
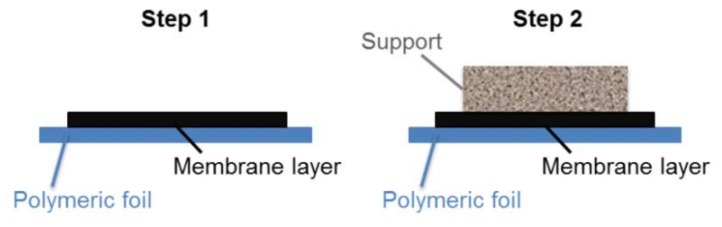
Schematic representation of the two-step sequential tape casting of asymmetric green tapes.

Sequential tape casting was used to fabricate asymmetric green tapes. The two-step sequential casting procedure is schematically depicted in Figure 1. First, the dense membrane layer was cast on a silicone-coated polymeric tape by a doctor blade with a casting gap of 50 µm. After drying at room temperature, the support slurry, containing the pore-forming agent, was cast directly on the membrane layer with a doctor blade gap of 1.9 mm. Finally, disk shape samples with diameters ranging from 24 to 30 mm were punched out from dried green tape.

Thermogravimetric analysis (TGA) was conducted on tape-cast green samples to investigate the burn-out behavior of the organic components. TGA measurements were performed on asymmetric samples (membrane + support) in a Linseis STA PT1600 thermobalance (Selb, Germany) up to 1000 °C using a heating rate of 2 K/min in air at atmospheric pressure.

Co-firing of the membrane and support layer was performed on disk-shaped samples at temperatures up to 1300 °C and dwell time of 5 h, in ambient air. Co-firing consisted of debinding, to burn out the organic components, and subsequent sintering, to achieve the desired microstructure. The debinding and sintering heating ramps were adjusted according to the arising defects on sintered membranes in order to obtain defect-free sintered samples.

### 2.3. Characterization of Asymmetric Membranes

To verify the microstructural parameters such as the support porosity and membrane layer thickness, Scanning Electron Microscopy (SEM FEG Tescan MIRA3) analysis was performed on both on the membrane surface and on polished cross-sections of embedded samples. Moreover, the support porosity was estimated by quantitative image analysis using Nikon LUCIA software (Tokio, Japan). SEM micrographs were converted into binary images by defining a gray-scale threshold, and the porosity was calculated as the ratio between the area of the masked regions and the total image area. The pore volume fraction estimated by this method corresponds to the total porosity (open + closed) of the layer.

The microstructure of the sintered samples was compared with an asymmetric LSCF reference membrane manufactured by tape casting [26]. 

The gas tightness of the sintered membranes was tested at room temperature by a helium (He) leakage test carried out by a Pfeiffer Vacuum Adixen ASM 340 leak tester.

High temperature (650–1000 °C) permeation tests were performed on asymmetric membranes in the experimental setup described in Section 2.1. The maximum leakage value of oxygen measured during high temperature tests was lower than 8%.

## 3. Results and Discussion

### 3.1. LSCF Powders Characterization

**Figure 2:**
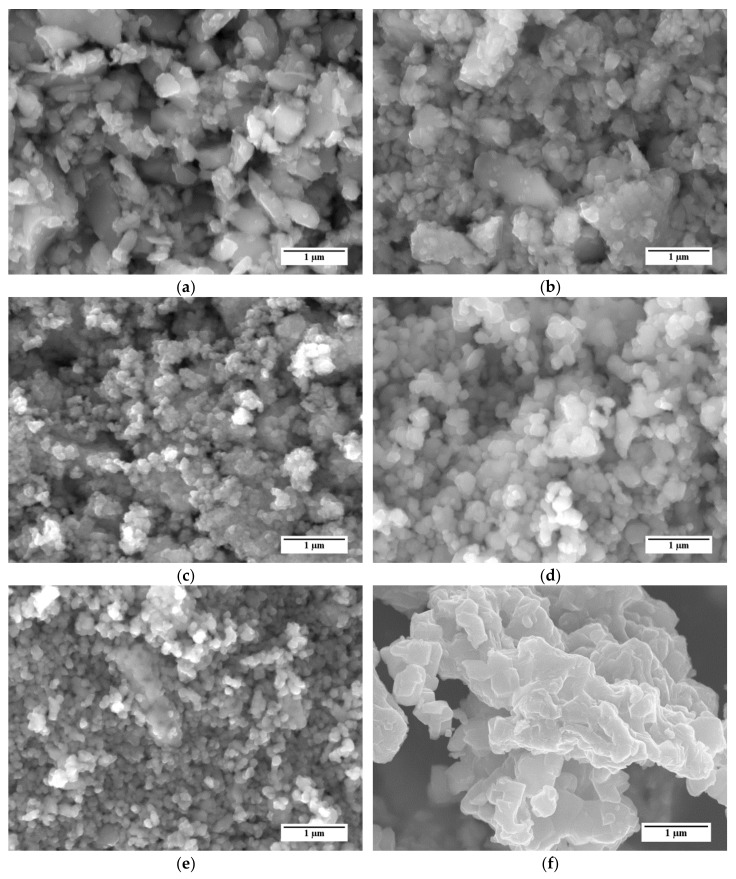
SEM micrographs of the La_0.6_Sr_0.4_Co_0.2_Fe_0.8_O_3−δ_ (LSCF) powders considered in this study: (**a**) Powder A; (**b**) Powder B; (**c**) Powder C; (**d**) Powder D; (**e**) Powder E; (**f**) Reference powder.

Representative SEM images of the different LSCF powders are shown in Figure 2. SEM micrographs reveal that all powders are mostly composed by partly sintered agglomerates of sub µm primary particles. For example, powders A and B (Figure 2a,b, respectively) show the presence of two main groups of particles, namely large grains with irregular shape and dimensions of few microns and smaller particles with dimensions of a few hundred nm. Powder C (Figure 2c) is almost completely composed by agglomerates of small particles with dimensions in the order of 100 nm. Samples of powders D and E (Figure 2d,e, respectively) qualitatively show a more homogeneous grain size distribution than samples A and B, which were characterized by a large presence of sub-micrometric particles and sporadic grains bigger than 1 µm. At a glance, the grains in powder E are smaller than those in powder D; this difference is confirmed by the granulometric analysis described below. 

For comparison, an SEM image of the reference LSCF powder is reported in Figure 2f. Similarly to the five LSCF powders considered in this work, this powder shows several agglomerates of particles. However, the agglomerates consist of larger particles, with dimensions exceeding 500 nm, with respect to the other LSCF powders.

The laser granulometry results and Brunauer–Emmett–Teller (BET) measurements are listed in Table 1. Powders consisting of agglomerates with sub-micrometer primary particles tend to have a higher surface area and internal porosity. Thereby, the specific surface area *A_spec_* is expected to increase with the decreasing primary particle size. The differences in the mean particle size of the five different LSCF powders are in good agreement with SEM observations (Figure 2). BET analysis points out very different surface area values (*A_spec_*) among the powders. Powder C shows the highest *A_spec_* despite a larger D_50_ value than powders B, D, and E. This is possibly due to the presence of a huge amount of agglomerates of sub-micrometer grains with a high inner porosity. Since the granulometric distribution is determined by laser diffraction, the determined D_10_, D_50_, and D_90_ values describe the dimensions of the agglomerates rather than the small primary particles. Therefore, a relation between grain size and specific surface area cannot be directly made.

**Figure 3:**
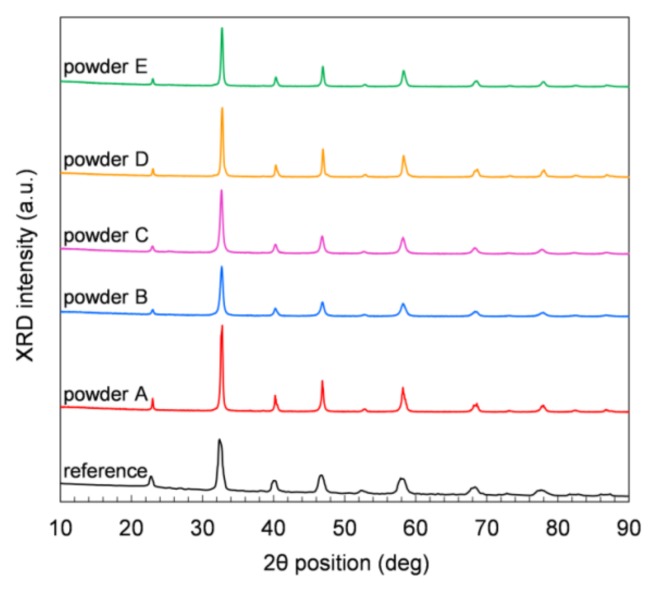
XRD patterns of the LSCF powders measured at room temperature.

Figure 3 displays XRD measurements performed on powder samples at room temperature (RT) in air. All the five commercial powders show comparable XRD patterns, and the positions of all peaks in each pattern match the rhombohedral perovskite structure of LSCF at RT (JCPDS chart No: 48-0124). No other crystal phases are observed in any sample. The Solvay reference powder (Figure 3) shows small amounts of orthorhombic and mainly rhombohedral perovskite structure. The orthorhombic phase can be explained by a too-short dwell time or temperature to form the rhombohedral perovskite structure. After heat treatment in air at 1200 °C, only the rhombohedral perovskite structure is obtained at RT.

ICP-OES measurements, listed in Table 2, confirm the aimed stoichiometry of all powders (La_0.6_Sr_0.4_Co_0.2_Fe_0.8_O_3−δ_).

**Figure 4:**
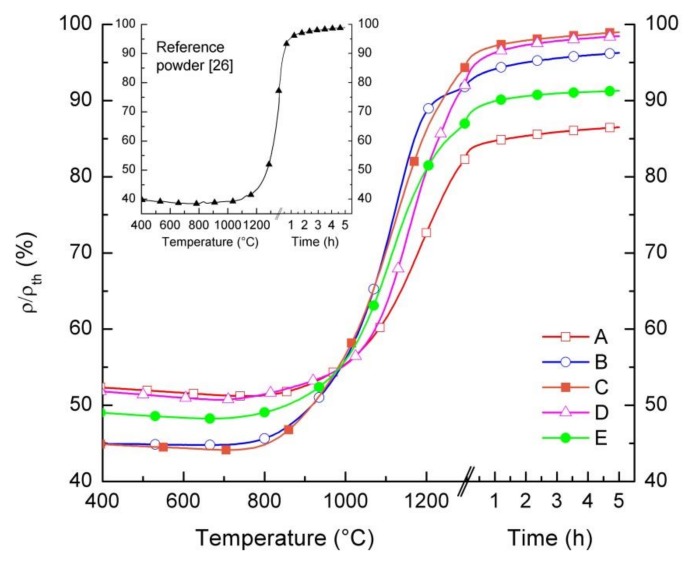
Relative density of pressed pellets prepared by the five LSCF powders as a function of temperature and dwell time. The inset shows the relative density of a pellet prepared by the reference powder, up to a temperature of 1350 °C with a dwell time of 5 h.

We studied the sintering behavior of the powders by differential dilatometric measurements. Pressed powder samples were heated up to 1300 °C with a heating rate of 5 K/min, while monitoring in real time the relative variation of the samples dimensions d*L*/*L*_0_. Figure 4 displays the relative density of samples as a function of temperature and dwell time. At a temperature below 750 °C, all samples, except for powder B, show a slight increase in dimensions due to the thermal expansion and, consequently, a slight density reduction (Δρ/ρ_th_ ≈ 1%). Starting from T = 750 °C, a gradual increase in relative density is observed in all samples, indicating that for such temperatures, the sintering of the grains starts to take place and persists up to 1300 °C. During the dwell time of 5 h at 1300 °C, only a slightly further densification occurs in any samples, ranging from 3% to 5%. This indicates that the maximum sintering activity is already obtained within the heating phase. After the dwell time, powders C and D achieve a relative density of 99% of the theoretical value. Lower relative densities are reached by the other powders, i.e., 87% for powder A, 96% for powder B, and 91% for powder E. We attributed the different relative densities after sintering to the different green densities and/or sintering activity of the powders.

The sintering behavior of the reference powder differs from the other powders. Due to the significantly lower surface area and larger particle size (cf. Table 1), densification starts at around 1100 °C, while the obtained relative density of the pellet is below 60% at 1300 °C without dwell time, when starting from a relative green density of 40%. To achieve a sufficiently high densification, it is necessary to increase the sintering temperature to at least 1300 °C, as reported in [26] and shown in the inset of Figure 4. 

Based on the sintering curves reported in Figure 4, the same thermal treatment (1300 °C/5 h) was identified for the five commercial powders to sinter pellets for oxygen permeation measurements.

**Figure 5:**
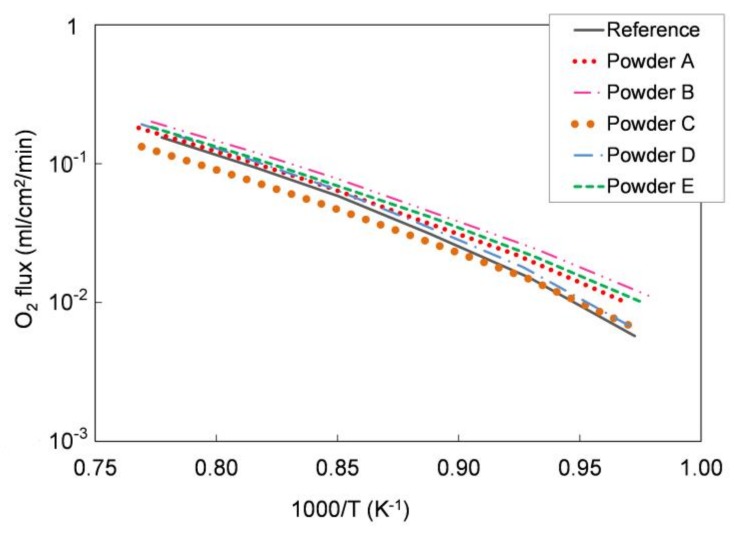
Oxygen flux normalized with respect to the process driving force as a function of temperature measured for pellets prepared with the five different LSCF powders, compared with that measured for a reference LSCF powder pellet. Samples thickness = 1 mm. Experimental error approx. 5%.

We tested the functional properties in an oxygen permeation test. Figure 5 shows the oxygen permeation rate as a function of temperature measured on the different pellets and compared with the oxygen flux measured for a pellet prepared with the reference LSCF powder. As reported in Section 2.1, the sample leakage during the measurements was always lower than 5.5% for all the tested pellets. As expected, the oxygen flux increases with the temperature in all samples and the permeation values are comparable for all powders. 

We further elaborated the permeation data and estimated the apparent activation energy of the permeation process for each sample. In the temperature range 750–1000 °C, the calculated values range from 117 to 137 kJ/mol for all the five powders, which is in good agreement both with the literature [28] and with the activation energy estimated for the pellets manufactured with the reference powder (139 kJ/mol). Hence, oxygen permeation tests highlight the comparable oxygen transport properties for all the examined LSCF powders.

Oxygen permeation data were used to calculate the ionic conductivities of the LSCF powders, according to the Wagner equation

(1) $$j_{O_{2}}=\frac{RT}{16F^{2}L}\int_{{p^{\prime }}_{O_{2}}}^{{p^{\prime \prime }}_{O_{2}}}\frac{\sigma_{el}\sigma_{ion}}{\sigma_{el}+\sigma_{ion}}d\ln p_{O_{2}},$$

where $j_{O_{2}}$ is the oxygen flux, *R* is the universal gas constant, *T* is the temperature, *F* is the Faraday constant, *L* is the membrane thickness, *σ_el_* and *σ_ion_* are the electronic and ionic conductivity, respectively, while ${p^{\prime }}_{O_{2}}$ and ${p^{\prime \prime }}_{O_{2}}$ are the oxygen partial pressure in the process side and in the permeate side, respectively.

At 900 °C, *σi_on_* is 0.073, 0.080, 0.053, 0.071, and 0.070 S·cm^−1^ for powders A, B, C, D, and E, respectively. Such values are comparable both with the conductivity of the reference powder (0.061 S·cm^−1^) and with the conductivity calculated from permeation tests on dense LSCF membranes previously reported (0.085 S·cm^−1^) [29].

The characterization of the five LSCF commercial powders confirmed equivalent crystal phase composition and stoichiometry, and comparable permeation performances. The most relevant differences were observed in particle average size and specific surface area values and, therefore, in sintering activity. Particle size and specific surface area are critical parameters for ceramic powders used to prepare slurries suitable for tape casting and have a strong influence on the whole manufacturing process. Therefore, the five powders were screened for tape casting on the basis of their morphological properties and the two powders with lowest and highest surface area—D and C, respectively—were selected for membrane preparation, in order to investigate the impact of powder morphology on slurries preparation and tape casting. The particle size of both powders is in the range 1–10 µm (cf. Table 1), which is appropriate for tape casting and avoids the sedimentation of large grains in the slurry [26]. The decisive main criterion for the selection is the very high densification (ρ/ρ_th_ = 99%) after sintering (Figure 4), which is an important requirement to obtain a fully dense membrane layer. In particular, the poor sintering properties are the main reasons why we did not consider powder A for the membrane realization, which has a comparable surface area with powder D. Indeed, the maximum relative density obtained after sintering for powder A (i.e., 87%) was significantly lower than the other powders (see Figure 4), and the presence of a high residual porosity in the membrane layer would preclude the gas tightness of the membranes.

The starting point for the investigation of the powder morphology on the tape-casting process of asymmetric membranes is the reference slurry recipe described in [19,26].

### 3.2. Slurry Formulation and Tape Casting

We prepared ceramic slurries for the membrane layer and support layer using powders C and D. Depending on the defects occurring after drying of the tape, the slurries’ composition was modified in order to avoid the corresponding defects. For this purpose, the amounts of solid loading and plasticizer I and II were adjusted according to the respective defect formation in the green tapes. The different slurry compositions are listed in Table 3.

The first slurry (*slurry 0*) realized with powder C, having a high surface area of 15.6 m^2^/g, was prepared according to the reference recipe (Table 3). However, the slurry had a sandy, solid appearance, so that it was not possible to cast it. Such behavior is due to the significantly higher surface area of powder C with respect to the reference powder (Table 1), because a larger amount of solvents is adsorbed inside the agglomerates porosity and on the grains’ surface. 

**Figure 6:**
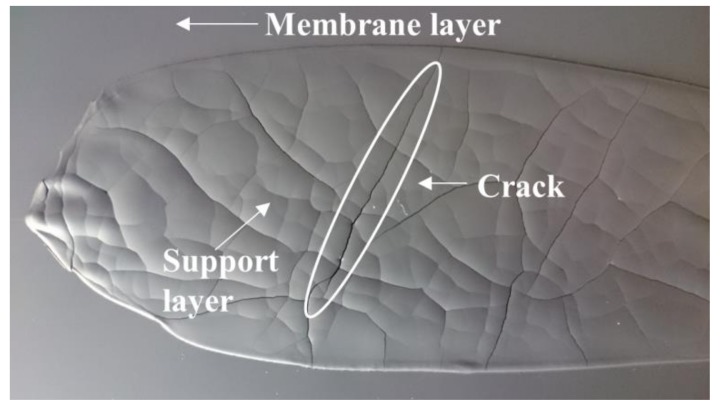
Green tape obtained by powder C after the sequential deposition of membrane and support layers using a reduced solid volume fraction (*slurry 1* recipe).

In such case, the fluidity of the slurry can be improved by increasing the volume ratio between the solvents and the ceramic powder. After reducing the solid powder fraction in both membrane and support slurries (*slurry 1*), it was possible to sequentially cast the two layers, according to the procedure represented in Figure 1. However, the high shrinkage caused by the large amount of solvents evaporation led to the formation of cracks in the thick support layer, which were cast with a blade gap of 1.9 mm, during drying (Figure 6). A similar cracking was reported by Schafbauer et al. and attributed to the brittleness of the green tape, due to a low binder content [25]. Moreover, the green tape must be adequately flexible and deformable to tolerate the dimensional change caused by shrinkage without breaking. Therefore, we increased the binder and plasticizer amounts (*slurry 2*) in order to obtain crack-free tapes. However, the suspension formulated with the extra quantities of binder and plasticizers was not sufficiently fluid for casting. Such results highlight that the optimization of the slurry composition for this powder is not straightforward and, due to the high surface area, powder C is not an appropriate choice for a reliable and reproducible fabrication of defect-free green tapes.

We observed a completely different behavior when we used Powder D, having the lowest surface area (Table 1). The slurries prepared with the reference recipes, both for membrane and support (*slurry 0*), showed a viscosity suitable for casting. However, after drying, a strong downward bending of the green tape and many pinholes in the membrane layer (not shown here) were observed. These pinholes were not present after the deposition and drying of the bare membrane layer. Casting the support on top of the dried membrane opened the pinholes, which became visible after drying the top cast support layer. We attributed their formation to the migration of solvents from support to the membrane layer through air bubbles, which were located at the interface between the membrane and support. 

**Figure 7:**
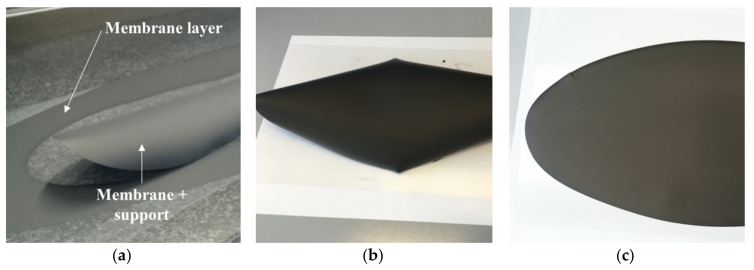
Green tapes obtained by using powder D after drying: (**a**) *Slurry* 1 (reference amount of PEG400); (**b**) *Slurry 2* (+100% PEG400); (**c**) *Slurry 3* (+167% PEG400).

In order to improve the bubbling up of air bubbles, which were entrapped while mixing, we increased the solvents’ volume fraction in the suspension (*slurry 1*), so as to facilitate the bubbles migration toward the slurry surface during the de-airing step. After casting and drying, no pinholes or other visible defects were observed on the membrane layer, but the tape bending was still present (Figure 7a). Such a reverse curling occurs in thick tapes and is mainly related to the fast solvent evaporation rate at the tape surface exposed to air [17]. Here, a dried thin layer is formed on the air-exposed surface, which hinders the solvent evaporation. In this situation, the solvent evaporation from the underlying regions is slowed down and the drying rate is significantly reduced, causing better rearranging of the particles during drying and therefore an increase in the total shrinkage. The different shrinkage from top to bottom causes a bending moment and a deformation of the tape, lifting it away from the polymeric carrier (Figure 7a).

Reverse curling can be avoided by promoting the relaxation of the internal stresses, which are formed in the tape during drying, through plastic deformation. The control of the solvents’ evaporation rate from the regions exposed to air is also important to preserve flatness. Both these effects can be achieved by using the proper quantities of organic components in the slurry. In particular, the addition of an extra amount of plasticizer II (PEG400) proved to have a favorable impact on the planarity of the tapes, as shown in Figure 7. In Figure 7b, the green tape obtained from *slurry 2*, where the PEG400 quantity was doubled with respect to the reference recipe, is reported. In comparison with the green tape from *slurry 1* (Figure 7a), the downward bending was mitigated by the extra plasticizer II amount but not completely suppressed. By further increasing the PEG400 amount in the suspension to +167% (*slurry 3*), the tape reverse curling was totally eliminated, and a planar, defect-free green tape was achieved (Figure 7c). The increase of the plasticizer II fraction has two main beneficial effects. First, it improves the flexibility of the polymer matrix in which the ceramic powder is embedded. Moreover, the extra amount that is not incorporated in the tape tends to segregate, since it has little chemical interaction with the other components, and forms a thin oily film on the surface exposed to the air, which slows down the solvents’ evaporation from the top regions and reduces the different drying rate from top to bottom.

**Figure 8:**
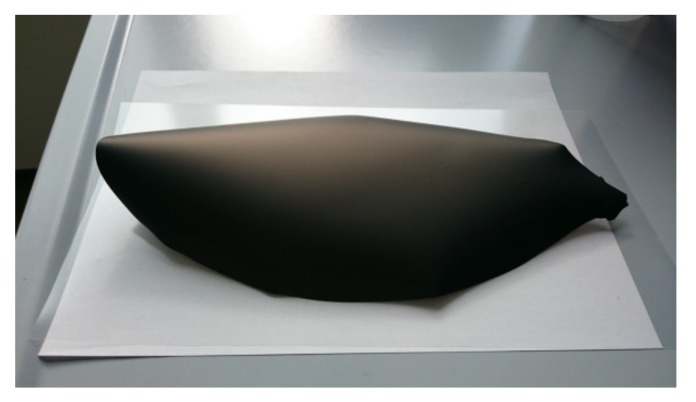
Green tape obtained by using powder D from *slurry 5* (+33% plasticizer I) after drying.

In addition, *slurry 4*, containing both a +167% amount of plasticizer II and a +33% amount of plasticizer I to improve the plastic deformation capability, resulted in a completely flat green tape (not shown here). The plasticizer I influence becomes obvious by adding a +33% extra quantity of plasticizer I and, at the same time, no extra amounts of plasticizer II (*slurry 5*). After deposition and drying, the green tape was curled in the edge regions, while the bending was significantly lower in the central part (Figure 8). This suggests that also the plasticizer I fraction in the slurry has a relevant influence on the reverse curling phenomenon and can be adjusted so to mitigate the possible tape bending.

To investigate the sintering behavior of all the tapes, we flattened curled green tapes after drying. Bending was eliminated by heating the curled tapes (i.e., obtained by *slurries 1*, *2,* and *5*) in a drying furnace above the glass transition temperature of the binder, to approximately 90 °C, and subsequently applying a uniform load on top of the tapes. After some minutes, the tapes recovered a flat geometry without any formation of cracks. The planarity was preserved after removing the load and cooling the tapes down to RT. Such a procedure is an effective solution to obtain small disk-shaped planar samples from these tapes; however, it cannot be considered an efficient processing to obtain large planar membranes from bended tapes, because it adds an additional handling step to the whole manufacturing chain. Moreover, it increases the probability of defects formation and potentially induces additional stresses to the tape. Therefore, for the scale-up of the tape-casting technology toward large-area components, slurries giving planar green tapes, such as *slurries 3* and *4*, are the most interesting and desirable.

### 3.3. Green Samples Debinding and Sintering

Disk-shaped samples were punched out from green tapes prepared using powder D for slurry compositions giving both planar tapes (*slurry 3* and *4*) and curled tapes after flattening (*slurry 1*, *2*, and *5*). 

**Figure 9:**
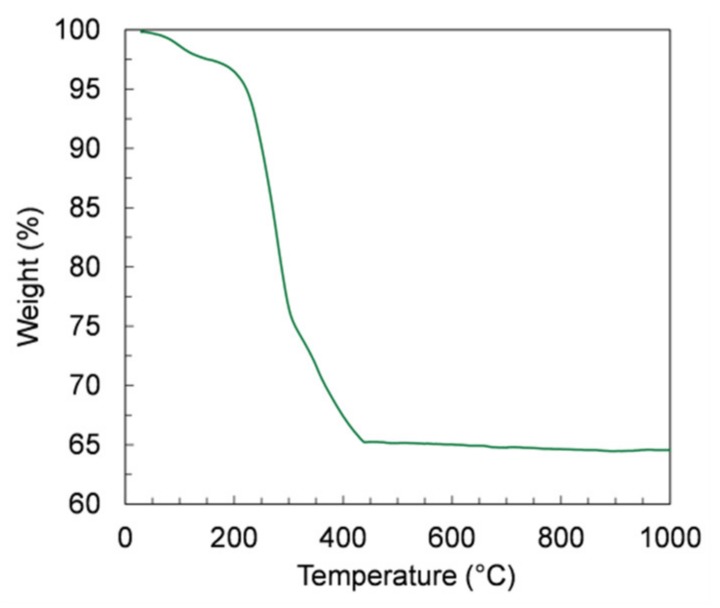
Thermogravimetric analysis (TGA) curve of the green tape prepared using powder D and *slurry 4*.

The debinding of the green samples is a critical step in the manufacturing, because a too-fast burn-out of the organic components can cause the release of a massive amount of energy and, consequently, the destruction of the thin membrane layer. Therefore, we investigated the organic release by TGA measurements on samples of green tapes prepared by *slurry 4*, which contain the highest organic amount. The TGA curve depicted in Figure 9 shows that the largest mass loss (34%) is observed from RT to 450 °C, corresponding to a massive release of organic components. At higher temperatures, the mass variation is negligible, confirming that the whole debinding process occurs at temperatures below 450 °C.

Based on dilatometric measurements results (Figure 4), we assumed a temperature of 1300 °C for sintering, with a dwell time of 5 hours. We initially chose a thermal treatment with uniform heating and cooling ramps of 2 K/min (referred to hereafter as *TT1*) for the co-firing of samples obtained from *slurries 1* and *2*.

As shown in Table 4 (*TT1* – red frame), for both slurry compositions, the sample planarity was preserved, but defects were formed. In particular, on samples obtained from *slurry 1*, circular and radial cracks were observed, while samples made with *slurry 2*, containing a higher organic fraction, showed the presence of both cracks and delamination.

Such defects are likely due to a fast evaporation rate of the organic components during the debinding step. Indeed, a too-fast pyrolysis of the organics components can cause uncontrolled sample combustion, which results in the damage of the membrane layer, as observed for *TT1*. In order to slow down the vaporization of the organics, we reduced the heating and cooling rates to 1 K/min. The samples obtained by this treatment, referred to as *TT2* (green frame), are shown in Table 4. In case of *slurries 1* and *2* being defect-free, almost planar sintered membranes were obtained, and the measured shrinkage was about 25%. On samples made with *slurry 5*, containing only a slight additional amount of organics, no visible defects were observed after co-firing (Table 4). Vice versa, samples obtained from recipes containing the highest organic fractions, i.e., *slurries 3* and *4* (see Table 3), showed a substantial upward bending and several cracks spreading across all the sample thickness after the treatment, indicating a spontaneous ignition of the organics. Therefore, despite allowing defect-free samples for some slurry compositions, *TT2* revealed to be not appropriate for those compositions giving planar green tapes after drying, i.e., *slurries 3* and *4*. 

**Figure 10:**
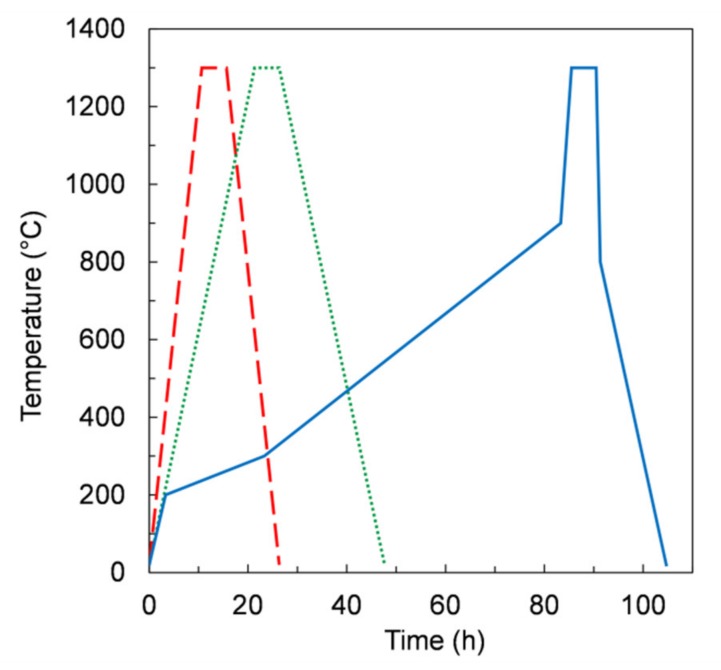
Temperature profile versus time in the debinding and sintering thermal treatments *TT1* (dashed line), *TT2* (dotted line), and *TT3* (solid line).

Aiming to control the burn-out process of organics, we further modified the thermal treatment. As reported in Figure 10, from RT to 900 °C, three segments with different heating rates were used. In particular, in the temperature range 200–900 °C the heating rates were lower than 0.2 K/min. The dwell time at 1300 °C for 5 h was kept unchanged. After this treatment, referred to as *TT3* (blue frame), the samples showed no defects for all slurry compositions (Table 4), including those giving planar tapes (i.e., *slurry* 3 and 4). After sintering, the samples were sufficiently flat for permeation measurement apart from the samples from *slurry 4* and *5*, which were slightly curved upwards. In these cases, planarity could be possibly improved by slightly changing the dwell time at 1300 °C, or alternatively the sintering temperature, in order to adjust the shrinkage of the porous support layer relative to the membrane layer, since the sintering activity is expected to be improved at higher temperature [21].

Such results confirm that depending on the slurry recipe, the thermal treatment must be consequently optimized in order to avoid the formation of cracks or other kinds of defects, which could be caused either by too-fast organic burn out or mismatched shrinkage of the membrane and support layer during the debinding and sintering processes.

### 3.4. Microstructure of Sintered Membranes

After debinding and sintering, we investigated the microstructure of the sintered samples to check the formation of the desired asymmetric structure. It is crucial that a compact and well-densified microstructure is achieved in the thin membrane layer, so as to guarantee the gas tightness of the membrane and produce high-purity oxygen. The porosity of the support layer is also an important parameter that has a strong influence on the membrane properties and must be carefully evaluated. Since the support layer is required to provide good mechanical stability, a low porosity would be desirable. On the other hand, the support layer causes resistance to the gas phase transport, and a low porosity can lead to strong oxygen concentration polarization in the support that has a detrimental effect on the permeation process [21,22,30]. To mitigate this problem, high-support porosity is required. Therefore, the porosity of the support layer must be a trade-off that is suitable to provide simultaneously high permeation performances and good mechanical resistance.

**Figure 11:**
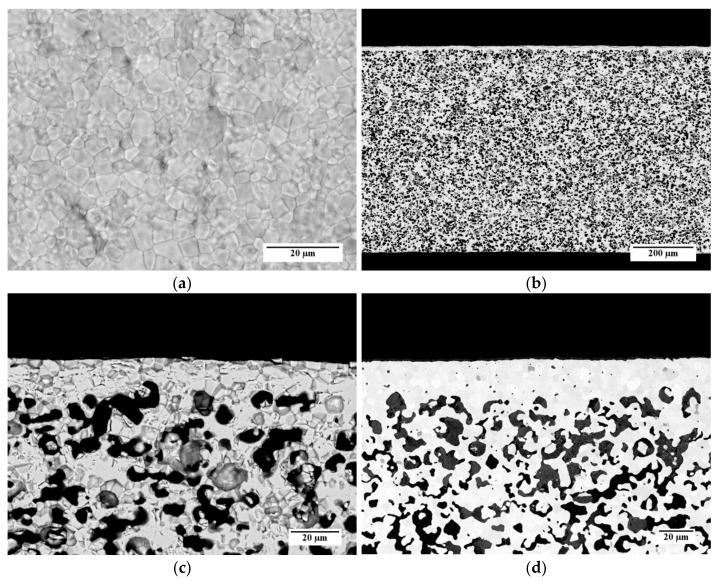
SEM micrographs of a membrane obtained using powder D from *slurry 3* and sintered by *TT3*: (**a**) Aspect of the membrane layer surface; (**b**) Cross-section; (**c**) Detail of the cross-section; (**d**) Detail of the cross-section of a membrane realized by the reference powder and recipe.

Figure 11 shows some representative SEM micrographs of a sample obtained from *slurry 3*, which is debinded and sintered by *TT3*. In Figure 11a, an SEM image of the top surface of the membrane layer is reported. The layer is characterized by a compact and dense microstructure made by sintered grains. The average grain size, estimated by the circular intercept segment method, is 3.1 µm. Few pinholes can be observed on the membrane surface (Figure 11a); however, the He leakage measurements carried out on this sample pointed out that the membrane was gas tight, with He-leak rates <10^−5^ mbar·L/(s·cm^2^). This indicates that such holes are superficial and their depth is not enough to pierce the dense membrane layer, or the pinholes are covered by the support material at the back side, respectively.

The cross-section of the whole membrane thickness (Figure 11b) confirms that the asymmetric membrane structure is obtained, consisting of a thin dense layer laying on a thick porous support. The support thickness is about 720 µm, and the porosity is uniformly spread across the whole layer. A higher magnification detail of the membrane cross-section is shown in Figure 11c. The sintered membrane layer is well densified and has a negligible residual porosity among the grains. The thickness ranges from 15 to 20 µm, as expected by using a 50-µm doctor blade gap for its deposition. The porous support is characterized by the presence of pores with different shapes and sizes, including circular pores with a diameter of few microns and elongated structures made of interconnected pores. Such a microstructure results from the burning out of the corn starch particles present in the slurry, which have dimensions in the range of 2–30 µm [31], and the shrinkage during the debinding and sintering steps. 

For comparison, the cross-section of a membrane realized by the reference powder and the reference slurry recipes for both membrane and support layers [26] is reported in Figure 11d. The microstructure is comparable with that of the sample prepared by powder D and the *slurry 3* recipe (Figure 11c); however, the membrane layer of the reference sample has a higher thickness of 22 µm on average. This is likely due to the higher solvent fraction used in the *slurry 3* with respect to the reference recipe, which causes a higher shrinkage of the green tape while drying. The pore size and shape are similar for both samples.

Quantitative image analysis was carried out on SEM cross-sections of the porous support to quantify the porosity. The calculated porosity of the support layer of the sample showed in Figure 11 is 35%, which liey in the 30–40% range reported as the optimum for asymmetric membranes [32,33]. Taking into account that this quantification procedure, as a statistical analysis, is affected by an uncertainty of 3–5%, the same values are obtained also for the other samples, prepared by the different slurry compositions and thermal treatments described above. This result is consistent with the fact that the same typology and fraction of pore former leads to similar porosities, while changing the organic content in the slurry and/or the debinding conditions has only a minor effect.

**Figure 12:**
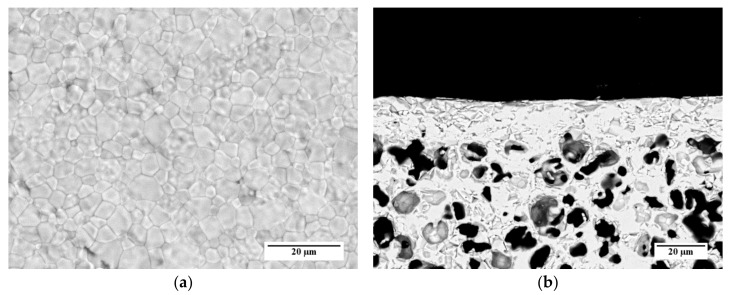
SEM micrographs of the membrane used for permeation tests, prepared by *slurry 1* recipe and sintered by *TT2*: (**a**) Aspect of the membrane layer surface; (**b**) Cross-section.

SEM investigations performed on samples prepared by the other slurry recipes (Table 3) and co-firing treatments (Table 4) point out that, in the investigated ranges, the membrane microstructure (i.e., grain size, membrane layer thickness, and porosity of membrane and support) is not influenced by the organic additive content and the heating rate. As an example, SEM images of the surface and the cross-section of a sample prepared by *slurry 1* recipe and debinded and sintered by *TT2* are reported in Figure 12a,b, respectively. The micrographs show a comparable microstructure with that of the sample obtained from *slurry 3* and *TT3* (Figure 11a–c).

**Figure 13:**
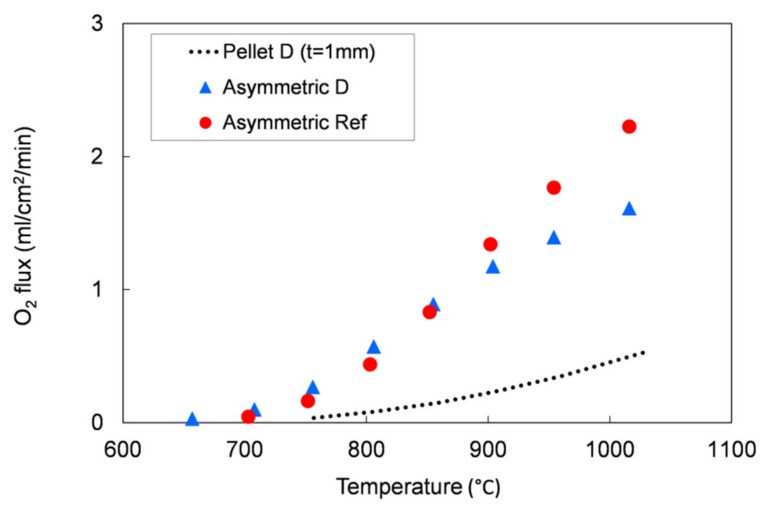
Oxygen flux as a function of the temperature. Results of permeation tests performed on an asymmetric membrane manufactured with the reference powder and on a pellet and an asymmetric membrane manufactured with the Praxair powder (D) selected in this work.

We evaluated the performances of the manufactured asymmetric membranes by high-temperature permeation tests, performed by feeding air on the support side of the sample and a sweep gas (Ar) on the membrane layer. The permeation tests were carried out on a membrane prepared by *slurry 1* recipe and debinded and sintered by *TT2,* which showed at room temperature He-leak rates <10^−5^ mbar·L/(s·cm^2^) and the lowest curvature after sintering. Additionally to the He-leak test, the membrane gas tightness was verified by continuously monitoring the nitrogen content in the permeate stream. The corresponding oxygen leakage was lower than 8% for the sample manufactured with the reference powder and lower than 4.5% for that manufactured with the Praxair powder (D) in the whole investigated operating conditions range, confirming the membrane density. The permeation test results were compared with the data obtained on the pellet prepared with powder D and on an asymmetric membrane manufactured with the reference powder (Figure 13).

As predicted by Equation (1), the oxygen flux rises with the temperature. The comparison between the 1 mm bulk and the asymmetric membrane samples manufactured with Praxair powder (D) confirms that the thickness reduction allows an increase of the membrane performance: at about 1000 °C, the permeation of the asymmetric membrane is around 3.2 times higher than the value measured on the pellet (thickness = 1 mm). At operation-relevant temperatures of 800 and 900 °C, the increase in oxygen flux is 7 and 4.9, respectively.

The permeation values for both asymmetric membranes are comparable at low temperature (T ≤ 850 °C), while at higher temperatures (850–1000 °C), the membrane manufactured from Praxair powder performs slightly lower than the sample made from the reference powder, despite the pellets showed comparable oxygen fluxes in the whole temperature range (Figure 5). This behavior can be attributed to a slight difference in the porous support thickness or morphology, as the gas diffusion pathways are also affected by tortuosity and pore opening diameter. Indeed, at low temperature, the oxygen transport through an asymmetric membrane is mainly limited by the surface exchange reactions, while at high temperature, the oxygen ion diffusion through the membrane and the gas transport in the porous support predominately control the oxygen permeation [7,22,32].

**Figure 14:**
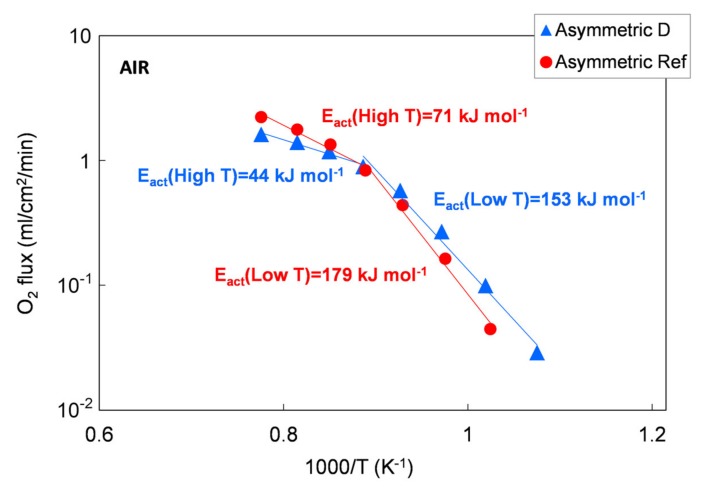
Oxygen flux as a function of temperature. Results of permeation tests performed on a membrane manufacture with the reference powder and with the Praxair powder (D) selected in this work.

The permeation results of asymmetric membranes are depicted in Figure 14 as an Arrhenius plot to highlight the change in the limiting transport mechanism. However, due to the porous support, the exact driving force across the membrane layer cannot be determined. Therefore, we used the permeation rate to calculate the apparent activation energies *E_act_* because a change in transport mechanism is still reflected by a change in *E_act_*, making a qualitative evaluation possible.

According to the literature, the apparent activation energy (*E_act_*) indicates changes in dominating transport steps, whereby the higher *E_act_* at temperatures below 850 °C can be attributed to predominant oxygen surface exchange [7,22]. At temperatures above 850 °C, bulk diffusion becomes rate limiting, which is in good agreement with the 1 mm bulk membrane measurement, where the *E_act_* is 117 to 137 kJ/mol for all the five powders in the temperature range of 750–1000 °C. A further decrease in activation energy indicates an increasing influence of the gas exchange inside the porous support, as reported in [32].

## 4. Conclusions

In this work, we investigated the influence of commercially available LSCF powders on the manufacturing of asymmetric LSCF membranes using tape casting. The following main conclusions can be drawn:

- A particle size in the range of 1–10 μm and a low surface specific area of the powder is inevitable for tape casting. These requirements were fulfilled by powders D and A.
- Powder D possesses one of the best sintering behaviors among all the powders.
- The slurry development was successfully done by using powder D. The final optimized co-firing step led to flat crack-free membranes. Nevertheless, this might be possible for other powders too, but this was not tested in this work. However, while limited modifications to the slurry composition were sufficient for powder D, a more complex slurry development is necessary for powders with higher specific surface area.
- The variation of the co-firing parameters (i.e., heating rates) showed no influence on microstructure (i.e., membrane layer thickness of 15–20 µm on a support with 35% porosity) and permeation properties (i.e., approximately 0.24 mL·cm^−2^·min for bulk and 1.2 mL·cm^−2^·min for the asymmetric membrane at 900 °C in air/Ar gradient) of membranes manufactured from powder D, compared to membranes manufactured from Solvay custom made powder, which was specifically designed for tape casting.
- The approach followed in this study may be also applied to other material systems, such as other MIEC or proton-conducting materials, since this approach was already successfully proven for BSCF [34] or LSCF [26] on larger scales.

## Figures and Tables

**Table 1:** Mean particle size (D_50_) and specific surface area (*A_spec_*) of the five LSCF powders.

Parameter	A	B	C	D	E	Reference
D_10_ [µm]	0.32	0.24	0.27	0.26	0.20	1.01
D_50_ [µm]	0.95	0.65	0.91	0.61	0.52	2.05
D_90_ [µm]	2.48	3.26	2.88	3.63	4.18	4.17
A_spec_ [m^2^/g]	3.7	10.3	15.6	3.5	7.4	1.5

**Table 2:** Stoichiometry of the LSCF powders, as determined by inductively coupled plasma optical emission spectroscopy (ICP-OES) measurements with a precision of 1–3% for major elements. For all values, the standard deviation is ≤0.02.

Powder	La	Sr	Co	Fe
A	0.60	0.387	0.197	0.81
B	0.598	0.387	0.204	0.811
C	0.618	0.394	0.195	0.793
D	0.62	0.39	0.256	0.73
E	0.618	0.393	0.201	0.788
Ref	0.602	0.395	0.203	0.801

**Table 3:** Composition of the different slurries prepared using LSCF powders C and D.

Slurry	Powder C		Powder D	
Slurry 0	Membrane:	Reference recipe [19,26,27]	Membrane:	Reference recipe [19,26,27]
	Support:	Reference recipe [19,26,27]	Support:	Reference recipe [19,26,27]
Slurry 1	Membrane:	−6 vol% powder fraction	Membrane:	−6 vol% powder fraction
	Support:	−6 vol% powder fraction	Support:	−6 vol% powder fraction
Slurry 2	Membrane:	−6 vol% powder fraction	Membrane:	−6 vol% powder fraction
	Support:	−6 vol% powder fraction +43% binder +43% plasticizer I +43% plasticizer II	Support:	−6 vol% powder fraction +100% plasticizer II
Slurry 3	------------		Membrane:	−6 vol% powder fraction
			Support:	−6 vol% powder fraction +167% plasticizer II
Slurry 4	------------		Membrane:	−6 vol% powder fraction
			Support:	−6 vol% powder fraction +33% plasticizer I +167% plasticizer II
Slurry 5	------------		Membrane:	−6 vol% powder fraction
			Support:	−6 vol% powder fraction +33% plasticizer I

**Table 4:** Sintered samples of asymmetric membranes obtained by using powder D for different slurry compositions and debinding and sintering thermal treatments. The sample diameter is in the range of 18–22 mm.

	Slurry 1	Slurry 2	Slurry 3	Slurry 4	Slurry 5
TT1	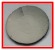	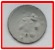			
TT2	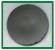	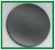	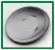	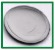	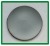
TT3	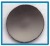	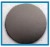	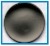	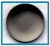	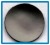
